# Evaluation of etanercept (a tumor necrosis factor alpha inhibitor) as an effective treatment for joint disease in mucopolysaccharidosis type I. A case report with whole-body magnetic resonance imaging

**DOI:** 10.3389/fmed.2023.1252704

**Published:** 2024-01-19

**Authors:** Natalia V. Buchinskaya, Eugenia A. Isupova, Anastasia O. Vechkasova, Damir A. Malekov, Dmitry O. Ivanov, Mikhail M. Kostik

**Affiliations:** ^1^Hospital Pediatry, Saint-Petersburg State Pediatric Medical University, Saint Petersburg, Russia; ^2^Radiology Department, Saint-Petersburg State Pediatric Medical University, Saint Petersburg, Russia; ^3^Neonatology Department, Saint-Petersburg State Pediatric Medical University, Saint Petersburg, Russia

**Keywords:** mucopolysaccharidosis type 1, MPS 1, glycosaminoglycans, arthropathy, etanercept, tumor necropsy factor-α

## Abstract

**Summary:**

A 12-year-old girl with mucopolysaccharidosis (MPS) type I (Gurler-Scheie syndrome, Q70X/del C683 of the IDUA gene in the compound heterozygous state) regularly received enzyme replacement therapy (laronidase) since the preclinical stage (6 months old) due to positive family history, and started etanercept treatment due to progression of joint pain and decreasing capability to walk. The patient had a significant reduction of pain in the joints and an expansion of daily physical activity without adverse events. A decrease in bone marrow edema without foci progression compared to baseline assessment was observed in the whole-body MRI.

During the treatment (baseline/6 months/12 months) the following was observed: childhood health assessment questionnaire (CHAQ) index of 1.88/2.13/1.63 points; patient’s pediatric quality of life inventory (PedsQL) of 37/30/31 points; parental PedsQL of 26/27/34 points; and patient’s pain visual-analog scale (VAS) of 75/45/40, with no VAS recorded for the mother. Juvenile arthritis functional assessment report (JAFAR) scores of 35/34/8 points were observed. A significant reduction in the taking of NSAIDs was observed. In the second half of the year, the nasal breathing became normal, and remission in chronic rhinitis and adenoiditis was achieved (no infection episodes) without otitis episodes.

**Conclusion:**

Etanercept in mucopolysaccharidosis type 1 is safe and well tolerated. The reduction of joint pain and increased walking capacity were observed. A decreased number of respiratory infection episodes and nasal breathing improvement were noted during the treatment. The observation shows the role of inflammation in the different aspects of MPS. Further investigations on immune system dysregulation in patients with MPS I are needed. Additional studies on the efficacy and safety of anti-rheumatic biological drugs in patients with MPSI are required.

## Introduction

Mucopolysaccharidosis (MPS) type 1 is an inherited metabolic disease characterized by a progressive course and multisystem manifestation. The disease is based on a deficiency of the alpha-L-iduronidase enzyme, which causes the accumulation of glycosaminoglycans (GAGs) dermatan sulfate and heparan sulfate in cell lysosomes (1). Several studies showing the role of GAGs in the development of inflammation have been previously published not only in MPS (2–4), but also in rheumatic diseases (5, 6). Joint involvement is one of the major problems for attenuated forms of MPS I, causing stiffness, contractures, and joint pain (7, 8). Despite the better outcomes of attenuated forms of MPS I compared to Hurler phenotypes, the delayed diagnostics and enzyme replacement treatment are still typical, and skeletal progression has not always been under the treatment control (1, 9). As inflammation might be one of the possible reasons for joint involvement and pain, anti-inflammatory drugs seem promising treatments (10–12). Etanercept is a biologic drug belonging to the family of tumor necrosis factor-α inhibitors and has been applied in the treatment of juvenile idiopathic arthritis for more than 20 years with high efficacy and a good safety profile (13, 14). A complete assessment of the quality of life and pain syndrome is possible only in children with attenuated forms of MPS and who have intact intellectual capabilities. Herein we describe the main changes in patient with attenuated MPS I during 12-month etanercept treatment.

## Case presentation

A 12-year-old girl with MPS type I (Gurler-Scheie syndrome, with two pathogenic variants Q70X/del C683 of the IDUA gene in the compound heterozygous state) regularly received enzyme replacement therapy (laronidase) since the preclinical stage from the age of 6 months due to positive family history. Her elder sister died at the age of 10 years from MPS I. The patient has had joint pains, and courses of non-steroidal anti-inflammatory drugs were prescribed.

Due to progressive pain, decreased range of motion, and quality of life, the etanercept treatment of 0.8 mg/kg/week was initiated at the age of 12 years. The study was approved by the Ethics Committee of Saint-Petersburg State Pediatric Medical University (protocol #1 from 19.01.2009) and by the local university authorities according to the national law. Written consent was obtained from the patient’s parents.

### Methods

For the assessment of treatment efficacy, we used data from physical examination, routine laboratory tests, whole-body magnetic resonance imaging (WB-MRI), and assessment of the quality of life and pain in children with the following questionaries: childhood health assessment questionnaire (CHAQ), the pediatric quality of life inventory (PedsQL), visual-analog scale (VAS), juvenile arthritis functional assessment report (JAFAR). Furthermore, we recorded data from the 6-min walking test and the number of days of taking non-steroidal anti-inflammatory drugs (NSAID) per month before etanercept onset and at 6 and 12 months of treatment. Adverse events during the 12-month treatment period were collected.

### Results

Before the start of etanercept treatment, the patient had the following complaints: persistent pain and stiffness of the joints, limited self-care, difficulty in walking long distances, fatigue, and increased pain after walking and exercising.

Joint status: all visible joints were deformed with a range of motion limitation, and the patient reported pain during movement examination, as well as stiffness.

WB-MRI showed the areas of trabecular bone marrow edema in the talus bones, in the femoral condyle. Synovial effusion in the ankle, knee, and hip joints was detected.

At the end of 12 months of weekly etanercept treatment, at the age of 13 years, no adverse events were noted. The patient noted a reduction of pain in the joints and the expansion of daily physical activity. During the examination, there were no significant changes in the joint status.

**Figure 1:**
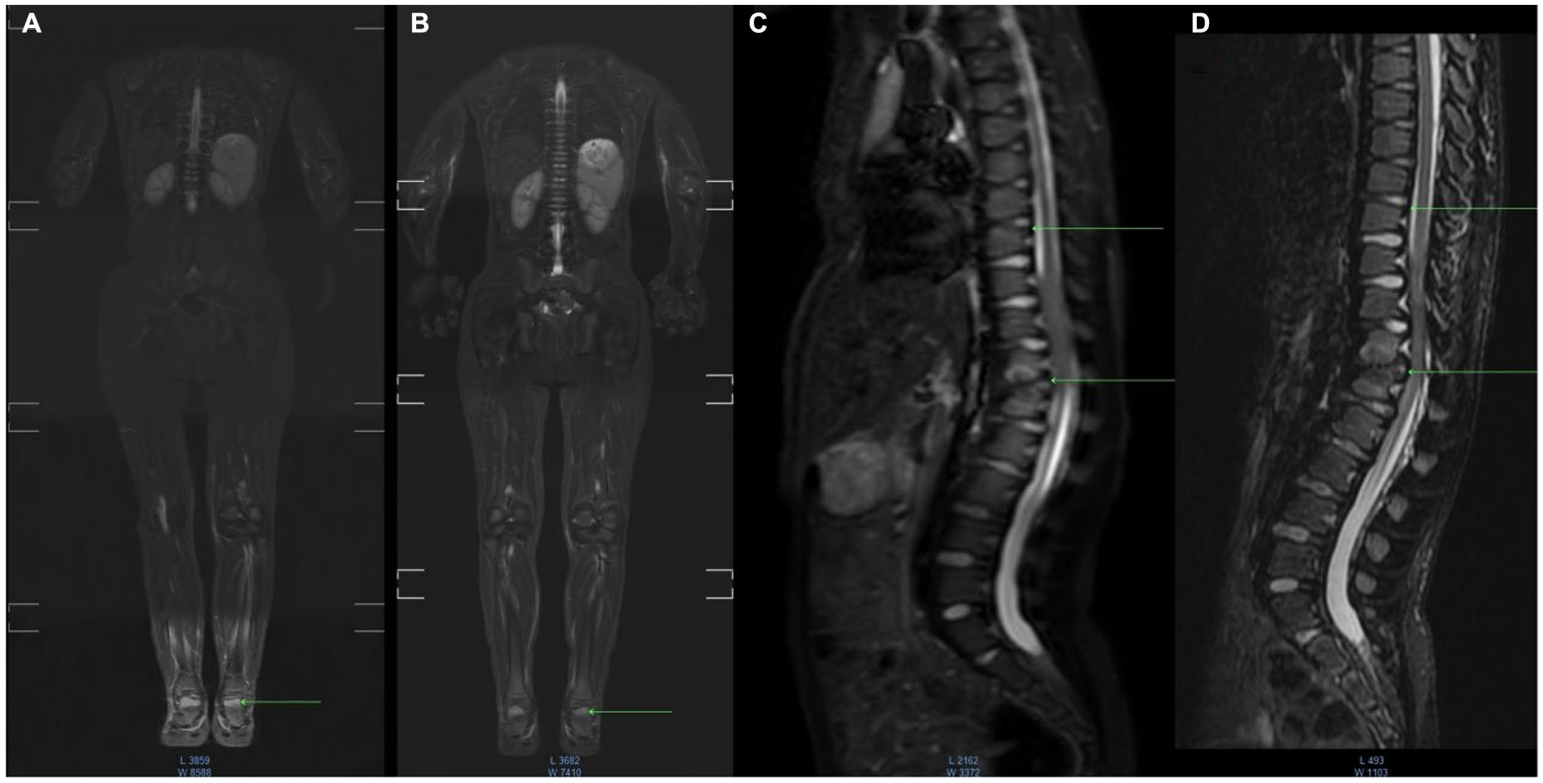
MR imaging dynamic research with fast spin-echo (SE) short inversion time inversion recovery STIR - fat suppression. Baseline or Inititial or Pre-etenercept treatment **(A,C)**; 12 months etanercept treatment **(B,D)** coronal whole body, sagittal images of the spine region. Image **(A)** shows swelling of the bones of the foot, in the dynamics of the next study **(B)** the intensity of edema decreased, and destruction in the foot was not detected. In the image of the thoracic spine in the upper vertebrae, there is swelling of the bone marrow **(C)**, whereas mechanisms of edema are not visualized **(D)** and the structure of the bone is not changed. In the lower Th12-L1 vertebrae, there is edema and areas of destruction of the vertebral bodies **(C)**, and mechanisms of edema showed that the intensity of edema decreased **(D)** and there were destruction zones without dynamics.

WB-MRI after 15 months of etanercept therapy showed a decrease of bone marrow edema foci intensity. No new foci of bone marrow edema and no progression of the size of the existing foci (Figure 1) were observed.

As for the quality of life assessment, some improvement in the indexes was observed during the treatment: CHAQ index before therapy was 1.88, whereas after 6 months of therapy, it increased to 2.13, and after 1 year of therapy, it was 1.63 points. The patient’s PedsQL, according to the child, was 37/30/31 points, and parental PedsQL was 26/27/34 points. The patient’s pain visual-analog scale (VAS) decreased in pain index: before/during/after, 75/45/40. We did not measure the mother’s VAS scores. The JAFAR index decreased from 35 points before etanercept treatment to 34 points and 8 points at the 6th and 12th month of treatment, respectively. A reduction in the taking of NSAIDs during the treatment with etanercept was observed. Since the 8th month of the treatment, the patient did not use NSAIDs. The data are shown in Table 1. In the second half of the year, the nasal breathing became normal, and remission in chronic rhinitis and adenoiditis was achieved (no infection episodes). There was no exacerbation in the ear (no otitis episodes and no hearing impairment).

**Table 1:** Quality of life assessment in the child with MPS I before and during the therapy with etanercept.

Assessments	Before etanercept	After 6 months of therapy	After 1 year of therapy
Age, years	12	12 and ½	13
CHAQ, points	1.88	2.13	1.63
PedsQL child, points	37	30	31
PedsQL mother, points	26	27	34
Patient’s visual-analog scale of pain, mm	75	45	40
The parental visual-analog scale of pain, mm	35	42	47
JAFAR points	35	34	8
6-min walk test, m	300	550	550
NSAID times per month, n	1–2	0.5	0

CHAQ, Childhood Health Assessment Questionnaire; JAFAR, Juvenile Arthritis Functional Assessment Report; PedsQL, The Pediatric Quality of Life Inventory; NSAID, non-steroidal anti-inflammatory drugs.

## Discussion

In the present case report, a successful treatment with etanercept was demonstrated in a patient with attenuated MPS I. The main benefits were: decreased joint pain, improvement in walking, and increased joint functional ability.

Osteoimmunology impairment and secondary inflammatory cytopathology are supposed to be the main cause of skeletal progression in MPS (11). For many years, MPS was supposed a non-inflammatory disorder, without any clinical and humoral signs of inflammation, but several studies have shown pro-inflammatory immune system dysregulation in patients with MPS (10). Raymond et al. showed a significant elevation of inflammatory markers including IL-1β, TNF-α, MCP-1, SDF-1α, IL-1Ra, MIP-1β, IL-8, and VEGF in the cerebrospinal fluid of patients with MPS I compared to unaffected children (11, 15). Van den Broek et al. demonstrated elevated pro-inflammatory blood profile in children with MPS before bone marrow transplantation (BMT) and 10 years of follow-up compared to children with phenylketonuria who had been in long-term remission due to an elimination diet. The majority of systemic markers associated with inflammatory status declined after BMT, but five markers of bone metabolism (RANKL, OPG, AXIN1, Flt3L, and SCF) persisted at highly elevated levels despite HST, and this might explain the ongoing skeletal disease after transplantation (16, 17).

L.E. Polgreen et al. described the role of TNF-alpha in inflammation and pain in various types of MPS (I, II, and VI). Their study, evaluating levels of TNF-alpha in the blood of 55 patients with MPS, as well as pain (measured with visual-analog scale) and functional disability (assessed with the children’s health questionnaire – Parent Form 50), showed a positive correlation between pain and functional disability and level of TNF-alpha. In addition, it was noted that the level of this cytokine does not significantly change in patients during enzyme replacement therapy or after BMT. The authors suggested TNF-alpha inhibitors as a possible additional treatment option for improving articular manifestations in patients with MPS (18).

The effectiveness of anti-TNF therapy was evaluated in a pilot 32-week, randomized, double-blind, placebo-controlled, crossover study of adalimumab conducted by L.E. Polgreen et al. in 2017. This treatment was considered safe and tolerable and could improve the range of motion, physical function, and possibly pain in children with MPS I (Hurler syndrome, after BMT) and MPS type II attenuated variant on ERT II compared to placebo (12).

Lund et al. showed that IL-6 and pyridinoline were the risk factors for the progression of joint contractures and hip dysplasia and linear growth delay in patients with mucopolysaccharidosis type I (19).

In our study, we chose etanercept based on previous data on the role of TNF-α in joint involvement in patients with MPSI, as well as on the biggest experience of this drug in pediatric rheumatology with confirmed efficacy and its very good safety profile (the lowest risk of the infections compared to other biologics) (13, 14, 20). In the last 10 years, trials of etanercept in juvenile idiopathic arthritis have demonstrated that the CHAQ score decreased from 0.8 (0.7; 0.9) to 0.2 (0.0; 0.5), pain score decreased from 51 (46; 55) to 7 (3; 11) mm, the number of active joints decreased from 6.7 (5.9; 7.6) to 0.2 (0.0; 0.5), and the number of joints with limited motion decreased from 5.7 (5.0; 6.5) to 0.5 (0.0; 0.9) (20). In our case, we did not observe such positive dynamics as in the patients with juvenile arthritis. The main positive changes were related to a decrease in the patient’s pain, improvement in the JAFAR index and 6-min walk test, and in the need for NSAID (20).

In our study, some efficacy of joint involvement was confirmed with clinical and radiological assessment. To the best of our knowledge, this is the first observation where whole-body MRI was applied to patients with MPS. Bone marrow edema (BME) is best investigated using fat-suppressed MRI T2W sequences. In the acute phase of bone injury by edema (Figures 1A,C), as well as in the subsequent phases of inflammation, the bone marrow underwent a remodeling process to fibrosis or connective tissue (Figures 1B,D).

During the study there were no side effects noted. Interestingly, the number of respiratory infection episodes decreased as the nasal breathing improved, which also might indicate immune-mediated inflammation and inflammatory-related hyperplasia of tonsils and adenoids in patients with MPS I.

The present case report has limitations related to a single case observation, no control group, and no possibility to assess the TNF-a and other pro-inflammatory cytokines.

## Conclusion

Etanercept in mucopolysaccharidosis type 1 is safe and well tolerated. The reduction of joint pain and increased walking capacity were observed. A decreased number of respiratory infection episodes and nasal breathing improvement were noted during the treatment. The observation demonstrates the role of inflammation in the different aspects of MPS. Further investigations on immune system dysregulation in patients with MPS I are needed. Additional studies on the efficacy and safety of anti-rheumatic biological drugs in patients with MPSI are required.

## Data availability statement

The original contributions presented in the study are included in the article/supplementary material, further inquiries can be directed to the corresponding author.

## Ethics statement

The studies involving humans were approved by the ethics committee of Saint-Petersburg State Pediatric Medical University (protocol #1 from 19.01.2009). The studies were conducted in accordance with the local legislation and institutional requirements. Written informed consent for participation in this study was provided by the participants’ legal guardians/next of kin. Written informed consent was obtained from the participant/patient(s) for the publication of this case report.

## Author contributions

NB, EI, AV, DM, DI, and MK were involved in the clinical care of the patient. NB, EI, AV, DI, and MK drafted the manuscript. DM drafted the Figure. All authors critically revised and approved the final version of the manuscript.
